# Receptor Tyrosine Kinases and Their Signaling Pathways as Therapeutic Targets of Curcumin in Cancer

**DOI:** 10.3389/fphar.2021.772510

**Published:** 2021-11-15

**Authors:** Sareshma Sudhesh Dev, Syafiq Asnawi Zainal Abidin, Reyhaneh Farghadani, Iekhsan Othman, Rakesh Naidu

**Affiliations:** Jeffrey Cheah School of Medicine and Health Sciences, Monash University Malaysia, Jalan Lagoon Selatan, Bandar Sunway, Malaysia

**Keywords:** curcumin, receptor tyrosine kinase, signaling pathway, polyphenol, combination therapy, tyrosine kinase inhibitor

## Abstract

Receptor tyrosine kinases (RTKs) are transmembrane cell-surface proteins that act as signal transducers. They regulate essential cellular processes like proliferation, apoptosis, differentiation and metabolism. RTK alteration occurs in a broad spectrum of cancers, emphasising its crucial role in cancer progression and as a suitable therapeutic target. The use of small molecule RTK inhibitors however, has been crippled by the emergence of resistance, highlighting the need for a pleiotropic anti-cancer agent that can replace or be used in combination with existing pharmacological agents to enhance treatment efficacy. Curcumin is an attractive therapeutic agent mainly due to its potent anti-cancer effects, extensive range of targets and minimal toxicity. Out of the numerous documented targets of curcumin, RTKs appear to be one of the main nodes of curcumin-mediated inhibition. Many studies have found that curcumin influences RTK activation and their downstream signaling pathways resulting in increased apoptosis, decreased proliferation and decreased migration in cancer both *in vitro* and *in vivo*. This review focused on how curcumin exhibits anti-cancer effects through inhibition of RTKs and downstream signaling pathways like the MAPK, PI3K/Akt, JAK/STAT, and NF-κB pathways. Combination studies of curcumin and RTK inhibitors were also analysed with emphasis on their common molecular targets.

## 1 Introduction

In 2020, The International Agency for Research Cancer (IARC) GLOBOCAN reported approximately 19.3 million new cases of cancer and 10 million deaths globally with data from 185 countries/territories. Lung, breast, and prostate cancers were the most commonly diagnosed cancers, while lung, liver, and stomach cancers were the most common causes of cancer death (Ferlay et al., 2021). Most cancer patients undergo combination treatments, for example, surgery combined with chemotherapy or radiotherapy. Chemotherapy alone can also consist of a combination or cocktail of drugs depending on the type and stage of cancer. Common chemotherapeutic drugs can be biochemically classified into alkylating agents (e.g. cisplatin, carboplatin, and etc.), anti-metabolites (e.g. gemcitabine, 5-fluorouracil), anti-tumour antibiotics (e.g. doxorubicin, epirubicin), topoisomerase inhibitors (e.g. etoposide) and tubulin-binding drugs (e.g. vinorelbine, paclitaxel, and doclitaxel) (Dickens and Ahmed, 2018). On the other hand, targeted therapy involves strategies that specifically target characteristic features in cells or proteins that enable cancer.

Receptor tyrosine kinases (RTKs) are a group of membrane-bound receptors that play an important role in the normal function of cells. They act as signal transducers that mediate cell-to-cell communication by phosphorylating tyrosine residues on key intracellular substrate proteins. Essentially, they lie at the centre of complex interconnecting signaling pathways and are actively involved in the maintenance of cellular homeostasis through regulation of cell proliferation, differentiation, metabolism, migration, and etc. (Wheeler and Yarden, 2015). Alteration or abnormal activation of RTKs have been recurrently observed and recognised as a contributing factor in the progression of various cancers (Weigand et al., 2005; Huang et al., 2011; Wang et al., 2011; Ha et al., 2013; Gallant et al., 2015). These observations led to the development of tyrosine kinase inhibitors (TKIs), which is a well-known targeted therapy. A commonly used TKI is the epidermal growth factor receptor (EGFR) tyrosine kinase inhibitors (TKIs) against non-small cell lung cancer (NSCLC). These small molecule inhibitors inhibit the tyrosine kinase domain of EGFR (Chan and Hughes, 2014). First- (gefitinib, erlotinib), second- (afatinib, dacomitinib), and third- (osimertinib) generation EGFR TKIs have been developed with slightly different mechanisms aimed at specific activating mutations (Lin et al., 2014). Other examples of TKIs and their targets include sorafenib (VEGFR kinase, RAF, PDGFR), crizotinib (ALK kinase), sunitinib (VEGF, PDGFR), imatinib (PDGFR, ABL kinase), carfilzomib (proteasome), ribociclib (CDK4, CDK6), and others. Despite their perceived efficacy, the use of TKIs are eventually met with the rise of resistance. Tumours either show a lack of response from the beginning of treatment or they slowly develop resistance after exposure to the drug (Simasi et al., 2014). RTKs mediate the emergence of TKI resistance through their oncogenic alterations such as mutations, overexpression, abnormal fusions and autocrine activation loops (Kobayashi et al., 2005; Cepero et al., 2010; Terai et al., 2013; Enrico et al., 2020). This poses a challenge to the clinical use of TKIs against cancer. Hence, recently, many researchers have begun studying the anti-cancer effects of naturally-derived compounds, mainly from the plant species. The idea behind this effort is to find an effective adjuvant that can be administered in combination with existing anti-cancer drugs, thus eliminating the common issue of toxicity associated with combination drug treatments.

Curcumin is a hydrophobic polyphenol extracted from the herb *Curcuma longa* or commonly known as turmeric. It was first introduced in 1910, but it has recently gained attention due to its potent therapeutic properties (Miłobȩdzka et al., 1910; Boroumand et al., 2018). Curcumin is a diferuloylmethane and its IUPAC name is (1E, 6E)-1,7-bis(4-hydroxy-3-methoxyphenyl)-1,6-heptadiene-3,5-dione (Giordano and Tommonaro, 2019). Various studies have shown that curcumin has anti-inflammatory (Farhood et al., 2019), anti-proliferative (Teiten et al., 2011), anti-oxidant (Boroumand et al., 2018), anti-microbial (Adamczak et al., 2020), anti-metastatic (Deng et al., 2016), and anti-angiogenic (Shakeri et al., 2019) properties. These properties altogether make curcumin a powerful anti-cancer agent. In India, where turmeric has been widely used as a cooking spice and medication for thousands of years, cancer rates are much lower compared to western countries. The lowest rates of cancer in India include esophagus, colorectal, liver, pancreas, lung, breast, uterine, ovary, prostate, bladder, kidney, renal, and brain cancers as well as non-Hodgkin lymphoma, and leukemia (Hutchins-Wolfbrandt and Mistry, 2011). There is however, a lack of hard evidence proving that turmeric consumption is solely or at least majorly responsible for the reduced cancer rates. Hutchins-Wolfbrandt and Mistry (2011) describe a few studies that have looked into daily turmeric consumption in India and Nepal, however, these studies did not examine how this affected the overall prevalence of cancer. Despite the lack of proven correlations, the availability and rapid expansion of curcumin-related research especially in the last decade, points towards its viability as an anti-cancer agent. Some of the main molecular targets of curcumin include transcription factors, growth factors, inflammatory cytokines, apoptotic proteins, protein kinases, receptors, cell survival proteins, microRNAs, tumour suppressor genes and oncogenes among others (Rahmani et al., 2014; Giordano and Tommonaro, 2019). In cancer, two of the most crucial roles of curcumin involves its ability to inhibit cellular proliferation and induce apoptosis. These features of curcumin target the root cause of cancer which is abnormal cell growth and apoptotic evasion. Several molecular targets of curcumin involving these two hallmarks of cancer are inhibition of growth factors and kinases (TGF-α, EGF, VEGF, FGF, FAK, JAK, MAPKs, mTOR, and etc.) and induction of apoptotic-related proteins (Bax, Bim, Bcl-2, Bcl-XL, and etc.) (Zhou et al., 2011). An essential component regulating these processes are RTKs and curcumin has been found to target RTKs like EGFR, VEGFR, FGFR, PDGFR, and others. Curcumin mainly downregulates RTK expression, inhibits RTK activation, decreases RTK ligands and also inhibits RTK downstream signaling pathways. More recently, several studies have also reported that curcumin enhances the effects of TKIs when administered in combination and in some cases, overcoming resistance altogether. The effects of curcumin are not only mediated through RTKs and involves many other components/molecular targets as mentioned before, however, RTKs seem to be at the core of these processes. Therefore, this current review discussed the role of receptor tyrosine kinases namely epidermal growth factor receptor (EGFR), vascular endothelial growth factor receptor (VEGFR), fibroblast growth factor receptor (FGFR), platelet-derived growth factor receptor (PDGFR), insulin-like growth factor 1 receptor (IGF-1R), and hepatocyte growth factor receptor (HGFR) in cancer and how curcumin targets these RTK signaling pathways including the mitogen-activated protein kinase (MAPK), the phosphatidylinositol 3-kinases (PI3K)/Akt, the Janus Kinase/Signal Transducer and Activator of Transcription (JAK/STAT) and NF-κB pathways. These RTKs were selected because they are well documented targets of curcumin in cancer. Additionally, drug combination studies involving curcumin and tyrosine kinase inhibitors were also reviewed with a particular focus on RTK inhibitors namely those targeting EGFR, VEGFR, and PDGFR.

## 2 Receptor Tyrosine Kinase Activation

### 2.1 Receptor Tyrosine Kinase Activation in Normal Cells

Tyrosine kinases can be further divided into receptor tyrosine kinases (RTKs) and non-receptor tyrosine kinases (NRTKs). Of all the 90 known tyrosine kinases, 58 are RTKs from 20 subfamilies (Table 1) while 32 are NRTKs from 10 subfamilies. All RTKs have a similar basic structure consisting of an amino terminal extracellular domain containing a ligand binding site, a single transmembrane α-helix, an intracellular tyrosine kinase domain, a tyrosine rich carboxy-(C) terminal and juxtamembrane regions (Lemmon and Schlessinger, 2010; Wheeler and Yarden, 2015).

**TABLE 1:** Classification of RTKs according to family.

Class	Family	Members
I	EGFR	EGFR, ERBB2, ERBB3, ERBB4
II	Insulin R	INSR IGFR
III	PDGFR	PDGFRα, PDGFRβ, M-CSFR, KIT, FLT3L
IV	VEGFR	VEGFR1, VEGFR2, VEGFR3
V	FGFR	FGFR1, FGFR2, FGFR3, FGFR4
VI	CCK	CCK4
VII	NGFR	TRKA, TRKB, TRKC
VIII	HGFR	MET, RON
IX	EPHR	EPHA1–6, EPHB1–6
X	AXL	AXL, MER, TYRO3
XI	TIE	TIE, TEK
XII	RYK	RYK
XIII	DDR	DDR1, DDR2
XIV	RET	RET
XV	ROS	ROS
XVI	LTK	LTK, ALK
XVII	ROR	ROR1, ROR2
XVIII	MUSK	MUSK
XIX	LMR	AATYK1, AATYK2, AATYK3
XX	Undetermined	RTK106

EGFR: epidermal growth factor receptor; InsR: insulin receptor; PDGFR: platelet-derived growth factor receptor; VEGFR: vascular endothelial growth factor receptor; FGFR: fibroblast growth factor receptor; CCK: colon carcinoma kinase; NGFR, nerve growth factor receptor; HGFR: hepatocyte growth factor receptor; EphR: ephrin receptor; Axl: from the Greek word anex-elekto, or uncontrolled, a Tyro3 protein tyrosine kinase; TIE: tyrosine kinase receptor in endothelial cells; RYK: receptor related to tyrosine kinases; DDR: discoidin domain receptor; Ret: rearranged during transfection; ROS: RPTK, expressed in some epithelial cell types; LTK: leukocyte tyrosine kinase; ROR: receptor orphan; MuSK: muscle-specific kinase; LMR: Lemur. Adopted from (Ségaliny et al., 2015).

RTK activation occurs through the binding of a ligand to the receptor, which then induces receptor dimerization. There are four general modes that have been proposed. These include 1) ligand-mediated dimerization, 2) ligand-mediated dimerization with receptor contacts, 3) ligand-mediated dimerization with receptors contacts and accessory molecules and lastly 4) receptor-mediated dimerization (Lemmon and Schlessinger, 2010). Once ligand-induced dimerization occurs, it activates the intracellular tyrosine kinase domain (TKD) through the transmembrane (TM) domain. The RTK TM dimer interface is very specific and contains essential structural information regarding the positioning of the catalytic domains. Interestingly, studies have found that switching the TM domains between different receptors can still result in constitutive activation as long as the catalytic domains are properly oriented (Cheatham et al., 1993; Petti et al., 1998; Li and Hristova, 2006).

Before TKDs are activated, each TKD is *cis*-autoinhibited by a specific group of intra-molecular interactions unique to each receptor. RTK activation occurs when this *cis*-autoinhibition is released after ligand binding and dimerization (Lemmon and Schlessinger, 2010). RTKs can be *cis*-autoinhibited by their activation loop, juxtamembrane region and C-terminal sequences (Mohammadi et al., 1996; Niu et al., 2002; Till et al., 2002). Transphosphorylation of tyrosine residues in each of these structures are required for activation. Certain TKDs can also be activated allosterically by their partners within a stable dimer. Autophosphorylation of RTKs occurs in several phases, with more tyrosine residues in the cytoplasmic region being autophosphorylated in a precise order (Lemmon and Schlessinger, 2010). The phosphorylated tyrosines or phosphotyrosines then become binding sites that recruit and assemble signaling molecules possessing the Src homology-2 (SH2) and phosphotyrosine-binding (PTB) domains. These specific molecules either bind directly to phosphotyrosine residues, or are indirectly recruited by binding to docking proteins phosphorylated by RTKs (Schlessinger, 2000). Some of these docking proteins include IRS1 (insulin receptor substrate-1), FRS2 and Gab1 (Grb-associated binder). These proteins further activate multiple downstream signaling pathways, with some of the main ones being the MAPK, PI3K, JAK/STAT, and PKC pathways (Lemmon and Schlessinger, 2010; Du and Lovly, 2018). These pathways regulate key processes such as survival, proliferation, differentiation, metabolism and cell-cycle control (Lemmon and Schlessinger, 2010). A more detailed account of these pathways will be included in the following sections.

### 2.2 Receptor Tyrosine Kinase Activation in Cancer Cells

**FIGURE 1:**
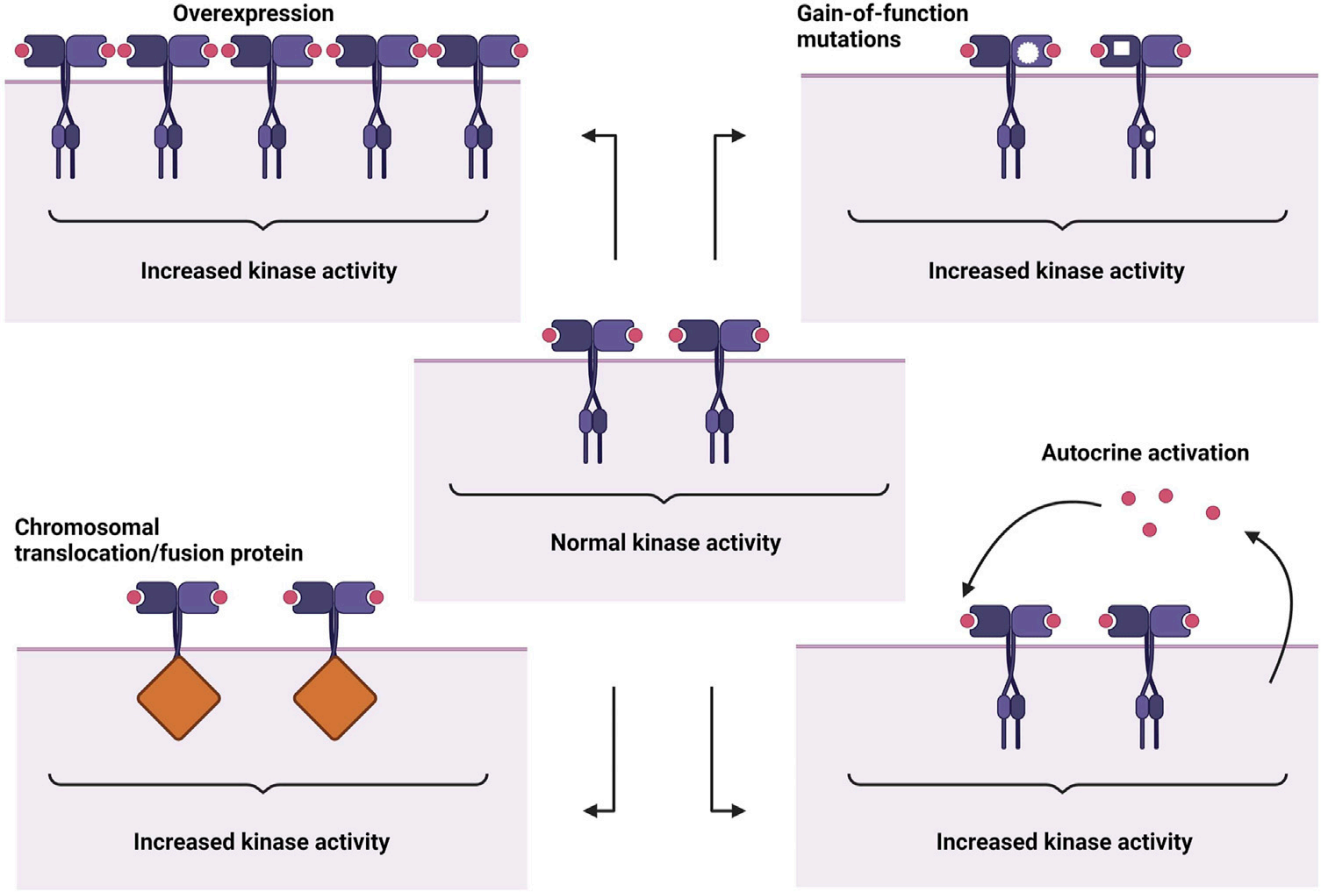
Abnormal RTK activation mechanisms. From top left: overexpression, gain-of-function mutations, autocrine activation, chromosomal translocation/fusion protein. Created with BioRender.com.

The abnormal activation of RTKs is a multifaceted process involving not just the RTKs themselves but also partner molecules and their surrounding environments. Their association with diverse groups of cellular components further complicates the mechanics of oncogenic RTK activation. Four main mechanisms leading to aberrant activation have been proposed (Figure 1), which are 1) RTK overexpression, 2) gain-of-function mutations, 3) chromosomal translocations, and 4) autocrine activation. In addition to these basic mechanisms, oncogenic RTK activation can also be influenced by kinase domain duplications, microRNAs, tumour microenvironment changes, negative RTK signaling regulators, protein tyrosine phosphatases, altered endocytic/trafficking genes and also spatial deregulation of RTKs (Casaletto and McClatchey, 2012; Du and Lovly, 2018).

#### 2.2.1 Epidermal Growth Factor Receptor

EGFR is an extensively studied RTK especially in lung cancer and aptly encapsulates the range of RTK oncogenic alterations. The best well-studied alterations are the EGFR activating mutations that occur in NSCLC. These mutations mainly occur in exons 18, 19, 20, and 21 of the TKD gene (Shigematsu and Gazdar, 2006). Approximately 90% of all EGFR activating mutations involve exon 19 deletions and the L858R point mutation. These mutations allow EGFR activation in the absence of ligand binding and shift the equilibrium between active and inactive states of the TK, enhancing kinase activity (Gazdar, 2009). Mutations also occur in the extracellular domain (ECD) of EGFR in lung (Yu et al., 2017), brain (Idbaih et al., 2009), and colon cancers (Arena et al., 2015). Some of these mutations were found to cause ligand-independent EGFR activation, EGFR amplification and disruption of anti-EGFR mAb binding.

EGFR amplification which commonly occurs due to mutation also occurs in breast (Shao et al., 2011; Park et al., 2014), lung (Morinaga et al., 2008), ovarian (Lassus et al., 2006), and prostate (Schlomm et al., 2007) cancers. The overexpression of EGFR leads to increased surface abundance which stimulates receptor dimerization and subsequent kinase activation (Casaletto and McClatchey, 2012). RTK surface abundance is also influenced by processes involving endocytic machinery and trafficking, whereby alterations of genes/proteins involved in RTK endocytosis can enhanced RTK activation (Bremm et al., 2008; Casaletto and McClatchey, 2012). EGFR gene fusions also occur in lung cancer and the most common fusion is EGFR-RAD51, which is a fusion between the EGFR TKD and RAD51, a DNA damage response protein (Konduri et al., 2016). EGFR-RAD51 can activate MAPK and PI3K/Akt pathways and promote cytokine-independent cell proliferation and colony formation, which are hallmarks of tumour cells (Konduri et al., 2016). Other EGFR fusions in NSCLC include fusions with purine-rich element binding protein B (PURB), septin 14 gene (SEPTIN14) and a recently discovered fusion partner, kinesin family member 5B (KIF5B) (Konduri et al., 2016; Zhu et al., 2019; Xu and Shao, 2020). EGFR can be abnormally activated through kinase domain duplications (KDDs) as well. EGFR-KDDs arise from in-frame tandem duplications of EGFR exons 18–25. Activation of EGFR-KDD occurs through the formation of ligand-independent intra-molecular dimers, which in turn amplifies signaling via ligand-dependent inter-molecular dimers (Du et al., 2021). EGFR-KDD has mostly been studied in lung cancer with regard to clinical outcomes (Wang et al., 2019; Chen et al., 2020). However the first case of EGFR-KDD was reported in a patient with esophageal squamous cell carcinoma, suggesting that it may be linked to hyper-progressive disease (Wang et al., 2020). Several studies also found that EGFR can be activated in an autocrine manner. Autocrine signaling occurs when both the target cell and secreting cell are the same cell. Autocrine signaling has been demonstrated to maintain cancer stem cells and also activate EGFR in tumour cells (Wu et al., 2007; Kim et al., 2012).

#### 2.2.2 Vascular Endothelial Growth Factor Receptor

There are three types of VEGFRs namely VEGFR1, VEGFR2, and VEGFR3 whereas there are five structurally related VEGF ligands including VEGFA, VEGFB, VEGFC, VEGFD, and placenta growth factor (PIGF) (Rapisarda and Melillo, 2012). There are also co-receptors involved in ligand binding called neuropilins (NRPs). Normal activation of VEGFRs generally lead to biological processes like angiogenesis, lymphangiogenesis, migration of endothelial cells, fatty acid uptake, etc. (Rapisarda and Melillo, 2012). VEGFRs and their ligands have been found to be expressed in lung (Tanno et al., 2004; Seto et al., 2006), breast (Filho et al., 2005; Zhao D. et al., 2015), colorectal (Lesslie et al., 2006), prostate (Chen et al., 2004), gastric (Yonemura et al., 2001) cancers. Overexpression is the most common mechanism of abnormal activation in VEGFRs. VEGFR1, and VEGF expressions were found to be elevated in pancreatic cancer cells leading to the activation of the MAPK pathway, which promoted cancer cell growth (Itakura et al., 2000). Meanwhile, an examination of 156 human gastric cancer specimens detected high expressions of VEGFR2, which correlated with poor overall survival (OS) (Lian et al., 2019). Using cDNA construct-transfected cells, they also found that VEGFR2 overexpression accelerated cell proliferation and increased cell invasive properties. VEGFR2 overexpression was also found in ovarian cancer cells (Spannuth et al., 2009), and it was linked to lower E-cadherin expression in breast cancer cells (Yan et al., 2015), suggesting its role in epithelial-mesenchymal transition. However, contradictory results were shown in a human carcinoid cell line whereby downregulation of VEGFR2 correlated to lower E-cadherin expression, suggesting the alternate roles of VEGFR2 in different cancers (Silva et al., 2011). The expression of VEGFR1, VEGFR2, and VEGFR3 has also been demonstrated to vary between the different stages of cervical (Van Trappen et al., 2003), prostate (Grivas et al., 2016) and ovarian (Klasa-Mazurkiewicz et al., 2011) cancers, with VEGFR3 being commonly overexpressed in the later stages. Autocrine activation of VEGFR occurs when VEGF ligands produced by the cancer cells proceed to activate the VEGFRs present on the same cancer cells. This autocrine feed-forward loop has been commonly demonstrated between VEGF:VEGFR2 (Jackson et al., 2002; Chatterjee et al., 2013; Song et al., 2019) and VEGFC:VEGFR3 (Kodama et al., 2008; Matsuura et al., 2009; Chen et al., 2010). Autocrine VEGF signaling also modulates treatment efficacy towards small molecule inhibitors in liver and gastric cancer, whereby higher expressions of VEGFR1/2 within the autocrine loop resulted in higher drug-induced inhibition of cell proliferation and delayed tumour growth (Peng et al., 2014; Lin et al., 2017).

#### 2.2.3 Fibroblast Growth Factor Receptor

There are seven types of FGFRs encoded by four different genes, which are FGFR1, FGFR2, FGFR3, and FGFR4. All of them have two different isoforms produced by alternative splicing except for FGFR4. On the other hand, over twenty FGFs can be grouped into seven families (Porta et al., 2017). FGFR signaling plays an important role during embryonic development and adult life. In cancers containing genetically altered FGFRs, the most frequent alteration can be found in FGFR1 (49%), followed by FGFR3 (23%), FGFR2 (19%), and lastly FGFR4 (7%) (Liu et al., 2021). The most common alteration is the amplification of FGFR genes and based on meta-analysis data, it mainly occurred in lung, breast, and gastric cancers (Chang et al., 2014). FGFR1 amplification was present in approximately 15–18% of lung squamous cell carcinoma patients as shown by three separate studies examining cases from 2000 to 2013 (Heist et al., 2012; Craddock et al., 2013; Monaco et al., 2015). FGFR1-amplified lung and breast cancer cells were shown to have enhanced activation of MAPK and PI3K signaling pathways, increased ligand-dependent signaling, and increased expression of stem cell markers (Turner et al., 2010; Ji et al., 2016). In gastric cancers, FGFR2 amplification was linked to poor progression free survival and overall survival (Matsumoto et al., 2012; Su et al., 2014; Hur et al., 2020), however it was also associated with high sensitivity towards FGFR inhibitors, suggesting the benefits of patient stratification based on FGFR amplification status (Xie et al., 2013; Pearson et al., 2016). Besides FGFR amplification, a comprehensive list of approximately 200 point mutations have also been found in FGFRs (Gallo et al., 2015). Mutations present in all four FGFR receptors were found in breast, colon, brain, lung and head and neck squamous cell carcinomas (Gallo et al., 2015). Acquired resistance to targeted therapies has also been linked to FGFR polymorphisms and gatekeeper mutations like V561M, leading to constitutive activation of FGFR1 (Cowell et al., 2017; Ryan et al., 2019; Mao et al., 2020). FGFR2 and FGFR3 are commonly involved in the formation of oncogenic gene fusions (Porta et al., 2017). The first gene fusion discovered was between FGFR3 and the transforming acidic coiled-coil containing protein (TACC3) forming FGFR3-TACC3 in glioblastoma (Singh et al., 2012; Parker et al., 2013). FGFR3-TACC3 fusions have been observed in lung (Capelletti et al., 2014; Wang et al., 2014), cervical (Carneiro et al., 2015), bladder (Nassar et al., 2018), and nasopharyngeal (Yuan et al., 2014) cancers and usually lead to increased cell proliferation, *in vitro* transforming abilities, and activation of MAPK and ERK signaling (Nelson et al., 2016). FGFR gene fusions usually involve partners possessing dimerization domains that allow ligand-independent receptor dimerization resulting in constitutive activation (Parker et al., 2014). Other FGFR gene fusions include BAG4-FGFR1, FGFR2-BICC1, FGFR2-CASP7, FGFR2-AFF3, and FGFR3–BAIAP2L1 (Wu et al., 2013).

#### 2.2.4 Platelet-Derived Growth Factor Receptor

There are two platelet-derived growth factors which are PDGFRα and PDGFRβ, also known as PDGFRA and PDGFRB. These two receptors are activated by five PDGFs which include PDGF-AA, PDGF-AB, PDGF-BB, PDGF-CC, and PDGF-DD (Fredriksson et al., 2004). Based on The Cancer Genome Atlas (TCGA) data, gene alterations in the PDGF family of ligands and receptors most commonly occur in lung cancer, colon cancer, and glioblastoma (Farooqi and Siddik, 2015). PDGFRB mutations in cancer have not been studied widely however, PDGFRA mutations are frequently observed in gastrointestinal stromal tumours (GISTs), especially in exon 18 (Heinrich et al., 2003; Daniels et al., 2011; Joensuu et al., 2015). PDGFRA and KIT mutations are associated with site and origin of tumours (Penzel et al., 2005), while gain-of-function mutation, V536E, led to increased phosphorylation of ERK and STAT5, causing constitutive receptor activation (Velghe et al., 2014). These mutations can occur on the regulatory domains (extracellular domain and juxtamembrane domain) or the enzymatic domain (tyrosine kinase domain (TKD)), which can lead to ligand-independent receptor dimerization or even kinase activation without receptor dimerization altogether (Lasota and Miettinen, 2006). Overexpression of PDGFRA mRNA has been recently observed in oral squamous cell carcinoma with links to metastasis and reduced patient survival (Ong et al., 2017; Ong et al., 2018). In lung cancer, ovarian cancer and medulloblastomas, PDGFR overexpression was associated with shorter overall survival, co-amplification with other RTKs and potential prognostic value (Lassus et al., 2004; Blom et al., 2010; Tsao et al., 2011).

PDGFR gene fusions are widely observed in hematological malignancies like acute myeloid leukemia, lymphoblastic leukemia and other myeloproliferative neoplasms (MPNs). PDGFRA fuses with five other intracellular proteins and one RTK, whereas PDGFRB fusions occur with twenty-nine other intracellular proteins (Appiah-Kubi et al., 2017). FIP1L1-PDGFRA is the most recurrent PDGFRA fusion gene that was first observed in patients with hypereosinophilic syndrome (Cools et al., 2003). Kinase activation of FIP1L1-PDGFRA is mediated by the disruption of the juxtamembrane domain of PDGFRA (Stover et al., 2006). Other gene fusion partners of PDGFRA include the breakpoint cluster region (BCR) (Yigit et al., 2015), KIF5B (Score et al., 2006), the CDK5 regulatory subunit associated protein 2 (CDK5RAP2) (Walz et al., 2006) and the ETS variant transcription factor 6 (ETV6) (Yoshida et al., 2015). As for PDGFRB, approximately 70 fusions have been identified with most fusion partners normally containing an oligomerization motif that mediates dimerization, causing continuous kinase domain activation in myeloid neoplasms (Campregher et al., 2017; Sheng et al., 2017; Xu et al., 2020). Autocrine PDGFR signaling plays an essential role in cancer progression in ovarian (Matei et al., 2006), breast (Jechlinger et al., 2006), thyroid (Adewuyi et al., 2018), and brain (Lokker et al., 2002) cancers. Both PDGFR and its ligands expressed in these tumours leads to an autocrine loop that fuels activation of downstream PI3K/Akt, MAPK and STAT pathways, in addition to the maintenance of EMT and enhanced metastasis (Jechlinger et al., 2006; Matei et al., 2006; Adewuyi et al., 2018).

#### 2.2.5 Insulin Receptor

The insulin receptor (IR) family consists of IGF-1R, IRA, IRB, IGF-1R/IR (hybrid), and IGF-2R. This review will focus on IGF-1R and IGF-2R, which are more frequently studied. IGF-1R is activated by ligands IGF-1 and IGF-2, while IGF-2R is a non-signaling receptor that mainly functions to clear IGF2 from the cell surface (Chen and Sharon, 2013). IGF-1R is overexpressed in a variety of cancers including colon (Shiratsuchi et al., 2011), pancreatic (Hakam et al., 2003), prostate (Aleksic et al., 2017), lung (Gong et al., 2009; Badzio et al., 2010), and breast (Jones et al., 2007) cancers. High levels of total IGF-1R were associated with higher tumour grade while higher levels of cytoplasmic IGF-1R were linked to a greater risk of post-radiotherapy recurrence in prostate cancer patients (Aleksic et al., 2017). Meanwhile, overexpression of IGF-1R in transgenic mice induces mammary tumour formation through activation of Akt, Erk1/Erk2, and STAT3 (Jones et al., 2007). Moreover, overexpression of IGF-1R also decreases tumour latency time, increases the proliferative genetic signature and enhances migration potential in mammary tumours in addition to protecting cells against stresses of the tumour microenvironment and apoptosis (Resnicoff et al., 1995; Valentinis and Baserga, 1996; Peretz et al., 2002; Ter Braak et al., 2017). IGF-2R has been found to be mutated in 60% of lung squamous cell carcinomas, while levels of IGF-2R appears to be much higher in the malignant stages of endometrial carcinomas (Kong et al., 2000; Pavelić et al., 2007). The existence of an autocrine loop was also found between IL-6 and IGF-1R whereby IL-6 induced expression of itself, forming a positive feedback loop further activating IL-6R, IGF-1R, IGF-1, and IGF-2 in NSCLC (Zheng et al., 2019). In acute myeloid leukemia, autocrine production of IGF-1 was shown to be responsible for the constitutive activation of IGF-1R and PI3K/Akt (Chapuis et al., 2010). Meanwhile, tumour cells have been proposed to secrete IGF-2 which binds to IGF-1R, increasing the rate of cellular proliferation through autocrine/paracrine signaling (Rasmussen and Cullen, 1998; Pavelić et al., 2003).

#### 2.2.6 Hepatocyte Growth Factor Receptor/C-Met

The hepatocyte growth factor receptor (HGFR), also known as MET or c-Met, is encoded by the MET gene, and its ligand is the hepatocyte growth factor (HGF) (Kumar et al., 2018). There is another Met-related RTK called Ron which binds to HGF-like protein/macrophage stimulating-protein (HGFL) (Wagh et al., 2008); however, this review will only focus on c-Met. C-Met has been found to be overexpressed in breast (Zhao et al., 2017), lung (Gumustekin et al., 2012; Awad et al., 2016), ovarian (Sawada et al., 2007), colon (Lee et al., 2018), cervical (Baykal et al., 2003), renal (Miyata et al., 2003), and blood (Jücker et al., 1994) cancers. Overexpression of HGF/c-Met was observed in NSCLC which led to subsequent lymph node invasion mediated by RhoA overexpression (Gumustekin et al., 2012). Mutations were also detected in the TKD of c-Met in this study; however, results suggested that it did not significantly impact RTK activation in NSCLC. In contrast, a more recent study reported MET exon 14 mutations that occurred predominantly in older patients with lung adenocarcinomas. Patients with advanced-stage NSCLC having these mutations also had concurrent MET gene amplification (Awad et al., 2016). A meta-analysis showed that c-Met overexpression correlated to distant metastasis, large tumour size and high histologic grade in breast cancer (Zhao et al., 2017). MET gene fusions have been primarily identified in lung cancer such as the HLA-DRB1-MET (Davies et al., 2017; Blanc-Durand et al., 2020), KIF5B-MET (Gow et al., 2018), MET-UBE2H (Zhu et al., 2018a), and MET-ATXN7L1 (Zhu et al., 2018b). The first case of HLA-DRB1-MET fusion was reported, however further studies are required to elucidate the specific mechanisms of the fusion gene (Davies et al., 2017). Meanwhile, it was also proposed that the MET-UBE2H fusion protein could be a novel resistance mechanism against EGFR-TKI treatment (Zhu et al., 2018a). MET fusions that occur at exon 15 and contain the 3’ MET kinase domain are thought to become activated due to constitutive dimerization of MET (Blanc-Durand et al., 2020).

Autocrine activation of HGF/c-Met signaling has also been described as a novel strategy leading to resistance against multi-kinase inhibitors in heptatocellular carcinoma (Firtina Karagonlar et al., 2016). Resistant cells were shown to have upregulated levels of HGF and activation of c-Met which when inhibited, resulted in lower migration and invasion capabilities (Firtina Karagonlar et al., 2016). Other studies examining hepatocellular carcinoma also found autocrine systems involving the Scatter factor (SF) and HGF/c-Met in metastasis as well as angiogenesis involving VEGF (Xie et al., 2001; Horiguchi et al., 2002). Autocrine activation of the MET receptor was also observed in colorectal cancer and acute myeloid leukemia involving components like β-catenin and co-activation of FGFR1 (Rasola et al., 2007; Kentsis et al., 2012).

## 3 Receptor Tyrosine Kinase Signaling Pathways Targeted by Curcumin in Cancer

**FIGURE 2:**
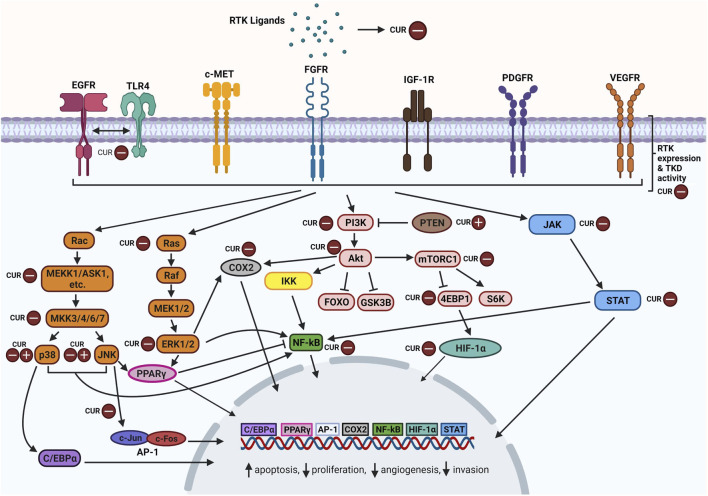
Overview of curcumin-inhibition of RTKs and downstream MAPK, PI3K/Akt, JAK/STAT, and NF-κB pathway components. Abbreviations: RTK: Receptor tyrosine kinase; CUR: Curcumin, EGFR: Epidermal growth factor receptor, TLR4: Toll-like receptor 4, c-MET: Mesenchymal epithelial transition factor/Hepatocyte growth factor receptor; FGFR: Fibroblast growth factor receptor; IGF-1R: The insulin-like growth factor 1 receptor; PDGFR: Platelet-derived growth factor receptor; VEGFR: Vascular endothelial growth factor receptor; TKD: Tyrosine kinase domain; Rac: Ras-related C3 botulinum toxin substrate; ASK1: Apoptosis signal-regulating kinase 1; C/EBPα: CCAAT/enhancer-binding protein alpha; JNK: c-Jun N-terminal kinases; AP-1: Activator protein 1; Ras: Rat sarcoma virus protein; Raf: Rapidly accelerated fibrosarcoma protein; MEK: Mitogen-activated protein kinase kinase; ERK: Extracellular signal-regulated kinase; PPARγ: Peroxisome proliferator-activated receptor γ; COX2: Cyclooxygenase-2; PI3K: Phosphoinositide 3-kinase; PTEN: Phosphatase and tensin homolog; Akt: Ak strain transforming; IKK: Inhibitor of nuclear factor kappa B kinase; NF-κB: nuclear factor kappa-light-chain-enhancer of activated B cells; FOXO: Forkhead box transcription factors; GSK3B: Glycogen synthase kinase 3 beta; Mtorc1: Mechanistic target of rapamycin complex 1; 4EBP1: Eukaryotic translation initiation factor 4E (eIF4E)-binding protein 1; S6K: Ribosomal protein S6 kinase; HIF-α: Hypoxia-inducible factor alpha; JAK: Janus kinase; STAT: Signal transducer and activator of transcription. Created with BioRender.com.

Activation of RTKs leads to a ripple effect whereby multiple signaling cascades are triggered to produce different outcomes. Curcumin has been found to modulate the expression of RTKs, their ligands and components particularly within their downstream MAPK, PI3K/Akt, JAK/STAT, and NF-κB pathways (Figure 2). Curcumin-mediated mechanisms mainly involve inhibition of specific components and in some cases, upregulation as well. Favourable outcomes have been observed in cancer cells following curcumin treatment including enhanced apoptosis, reduced cellular proliferation, reduced angiogenesis and reduced migration. Curcumin appears to act as a tyrosine kinase inhibitor as reviewed by Golonko et al. (2019) and Farghadani and Naidu (2021), similar to the mechanism of TKI drugs but with more pronounced effects. In this section, we take a closer look at how curcumin modulates these signaling pathways.

### 3.1 Effects of Curcumin on Receptor Tyrosine Kinase Signaling Pathways in Cancer

#### 3.1.1 Mitogen-Activated Protein Kinase

The mitogen-activated protein kinase (MAPK) pathway is one of the main pathways involved in the regulation of cellular proliferation, differentiation, development, apoptosis, and transformation. Three MAPK families have been well characterised which include extracellular signal-regulated kinase (ERK), Jun kinase (JNK) and p38 kinase. MAP kinases are activated in a cascade fashion following stimulation by growth factors, cytokines, stress and ceramides among others. A MAP kinase cascade is normally a series of activations involving a MAPK kinase kinase (MAPKKK), a MAPK kinase (MAPKK), and a MAP kinase (MAPK) (Zhang and Liu, 2002). The Raf-MEK-ERK is one of the well characterised MAPK signaling pathways. The multistep process that occurs after RTK activation starts with recruitment of adaptor proteins (Grb2, Sos, etc.) followed by activation of c-Raf (MAPKKK), MEK1/2 (MAPKK) and finally ERK1/2 (MAPK). ERK then translocates to the nucleus and phosphorylates transcription factors like the ternary complex factor (TCF) Elk-1, c-Myc, serum response factor accessory protein Sap-1a, Ets1, Tal, and others (Zhang and Liu, 2002). The JNK proteins also known as stress-activated protein kinases (SAPKs) are primarily activated by stress conditions like DNA damage, UV irradiation, and inflammation (Katz et al., 2007). Growth factors like EGF, PDGF, and FGF are less efficient stimulants (Kyriakis and Avruch, 2001). JNKs are directly phosphorylated by MKK4 and MKK7 (MAPKKs) while these MAPKKs are dually phosphorylated by MAPKKKs which include the MEKK family, the mixed-lineage kinase family, the apoptosis signal-regulating kinase family, TAK1 and TPL2 (Davis, 2000). Upon activation, JNKs can activate a range of proteins including the activator protein-1 (AP-1) which is formed through the dimerization of Jun (c-Jun, JunB, and JunD) and Fos (c-Fos, FosB, Fra-1, and Fra-2) proteins. Lastly, the p38 kinase has four isoforms, α, β, γ, and δ (Canovas and Nebreda, 2021). They can be phosphorylated by the MAPKKs, MKK3, MKK4, and MKK6. Prior to this, these MAPKKs are phosphorylated by MAPKKKs such as ASK1, DLK, MEKK3, MEKK4, TAK1, and etc. Overall, growth factors mainly activate the ERK1/2 cascade, partially activate JNK, and rarely activate p38 (Katz et al., 2007). Hence, this review will mainly focus on the ERK pathway.

In cancer, constitutive activation of ERK signaling is normally caused by RTK overexpression and activating mutations in RTKs or components like Ras or B-Raf (Dhillon et al., 2007). Activating mutations in K-Ras and N-Ras have been observed in many cancers and commonly lead to inefficient GTP hydrolysis, leaving Ras in a constantly active, GTP-bound state (Dhillon et al., 2007; Prior et al., 2020). There are three isoforms of Raf namely, Raf-1/C-Raf, B-Raf, and A-Raf which are direct effectors of Ras. B-Raf gene mutations are present most commonly in melanoma (40–70%) and to a lesser extent in thyroid, colorectal and ovarian cancers (Rahman et al., 2013). The missense mutation, V600E, is the most common B-Raf mutation (∼90% of cancers) that results in constitutive activation of the MEK-ERK pathway without external stimuli, causing uncontrolled cellular proliferation (Cantwell-Dorris et al., 2011). C-Raf and A-Raf mutations are quite rare and it was found that C-Raf had a low basal kinase activity, which may explain its weak oncogenic effect (Emuss et al., 2005). MEK1/2 mutations are rare as well and they are mainly influenced by upstream mutations in Ras/Raf. Lastly, mutations in ERK were found to confer resistance to ERK and Raf/MEK inhibitors by disrupting drug binding and maintaining levels of ERK activity in B-raf mutant melanoma cells (Goetz et al., 2014). Oncogenic activation of JNK1 and JNK2 have been found in liver (Chang et al., 2009), pancreatic (Tian et al., 2021), bladder (Pan et al., 2016), and gastric (Mishra et al., 2010) cancers. The JNK pathway also promotes cancer cell survival via autophagy involving Bcl-2, tumour immune evasion, compensatory cell proliferation, and interaction with other signaling components such as NF-κB, p38, and JunD (Wu Q. et al., 2019). As for p38, it also acts as a tumour suppressor and inhibition of p38 mediates Ras-induced transformation (Dhillon et al., 2007). Tumour sizes have been found to be inversely correlated to p38 activity in hepatocellular carcinoma (Iyoda et al., 2003). Oncogenic MAPK signaling activated by RTKs are found in various cancers (Wu et al., 2006; Wang et al., 2012; Tang et al., 2017; Jiang et al., 2020).

In lung cancer cells, two separate studies examined the effects of curcumin on RTKs and their pathway components (Lev-Ari et al., 2006; Lev-Ari et al., 2014). Both studies found that curcumin downregulated expressions of COX-2 and *p*-ERK1/2 in a dose-dependent manner, however only one study observed downregulation of EGFR (Lev-Ari et al., 2006). EGFR signaling has been shown to induce transcription of COX-2 likely through the activation of MEK/ERK pathway, which explains the simultaneous downregulation of these components by curcumin resulting in decreased survival and enhanced apoptotic effects (Huh et al., 2003; Chi et al., 2016). Curcumin also showed dose-dependent inhibition of MyD88, TLR4, and EGFR in lung cancer cell lines (Zhang et al., 2019). Studies have found that TLR4 requires EGFR to signal and activation of TLR4 has also been linked to the MAPK pathway (Qian et al., 2008; De et al., 2015). It is possible that curcumin indirectly modulates the MAPK pathway by synergistic targeting of EGFR and TLR4. In addition, this study also found that curcumin lowered the expression levels of c-Jun and c-Fos proteins which make up AP-1, a major target of JNK. This led to decreases in other cell cycle proteins like cyclin A1, cyclin B1, cyclin D1, and etc. suggesting curcumin’s role in regulating cell cycle transitions via MAPK signaling activated by RTKs (Zhang et al., 2019). Another study found that curcumin inhibited VEGF and a wide range of downstream MAPK-related components including c-Jun-p, Ras, Grb2, MEKK3, and MKK7, however, levels of JNK and ERK seemed to be upregulated (S. S. Lin et al., 2009). JNK 1 and 2 have been observed to have opposing functions (Yin and Yang) in cellular environments (Wu Q. et al., 2019). Studies have found that JNK1 mediates cell survival while JNK2 contributes to apoptosis however, the opposite has also been observed (Arbour et al., 2002; Liu et al., 2004). Hence, through inhibition of VEGF, curcumin may have indirectly upregulated the pro-apoptotic JNK protein levels since JNK is not a direct target of curcumin (Chen and Tan, 1998; S. S.; Lin et al., 2009). Lastly, the effects of curcumin was examined in an *in vivo* cancer model involving transgenic mice expressing VEGF-A (Tung et al., 2011). Curcumin significantly downregulated levels of VEGF protein and also mRNA levels of *vegf, vegfr2 (kdr), nrp-1, egfr,* and *erk2*. *Nrp-1* is the co-receptor of *vegfr2* and it was suggested that curcumin-induced downregulation of its downstream pathways, resulted in reduced VEGF expression. This was also evidenced by downregulation of *erk2*, reaffirming that the MAPK pathway plays a role in curcumin-mediated RTK inhibition (Tung et al., 2011).

In colon cancer, curcumin downregulated the EGFR gene expression by suppressing the early growth response-1 (*egr-1*) gene and the transactivation activity of Egr-1, a transcription factor that binds to the *egfr* promoter (Chen et al., 2006). Suppression of *egr-1* gene by curcumin was via disruption of ERK signaling which led to a decrease in Elk-1 phosphorylation (Chen et al., 2006). Besides, curcumin also suppresses EGFR expression by activating PPARγ in colon carcinoma cell lines (Chen and Xu, 2005). PPARγ can be inactivated through phosphorylation by ERK and/or JNK. It was found that curcumin-induced inhibition of MAPK activity led to increased activation of PPARγ and subsequent EGFR gene downregulation (Chen and Xu, 2005). Meanwhile, treatment of colon cancer cells with curcumin or dasatinib induced significant reduction of *p*-EGFR while combination treatment led to much greater reduction of both *p*-EGFR and *p*-IGF-1R (Nautiyal et al., 2011). Accordingly, downstream *p*-ERK1/2 levels were also reduced by a larger magnitude after combination treatment which may have resulted in the reduction of COX-2 levels that was observed as well. In colon cancer cells, after 3 h of exposure to high concentration of curcumin (100 μmol/L), a cDNA microarray analysis showed that levels of MAPK-related genes like MAP3K10 and MAP4K2 and also VEGF and FGFR1 were upregulated (Van Erk et al., 2004). Several other MAPK genes like MAP2K2 and MAPK8 were downregulated after exposure to low concentrations of curcumin (30 μmol/L) for 3 h. The high concentration of curcumin used in this study was found to decrease the cell number and result in floating cells hence, the several unexpected gene expression changes observed could be toxic-related effects of curcumin (Van Erk et al., 2004).

In breast cancer cell lines that overexpress HER-2 (BT-474 and SK-BR-3-h), curcumin downregulated the HER-2 oncoprotein and also the phosphorylation of MAPK in a dose-and time-dependent manner (Lai et al., 2012). Meanwhile, in triple negative breast cancer (TNBC) cells curcumin did not alter the expression of EGFR and ERK1/2 however, it significantly reduced the levels of phosphorylated EGFR and ERK1/2, showing that it specifically inhibits activation of EGFR and its downstream signaling molecules to reduce cell proliferation (Sun et al., 2012). Curcumin was also found to reduce EGFR activation and EGF-induced phosphorylation of ERK1/2 as well as JNK activity in breast cancer cells however, there was a lack of inhibition of p38 (Squires et al., 2003). This provides evidence that curcumin inhibition occurs via RTK signaling pathways since RTKs mainly activate ERK and JNK (partially) and p38 to a much lesser extent.

Using pancreatic cancer cells, it was shown that curcumin reduced hyperglycemia-driven EGF-induced metastatic abilities (Li W. et al., 2019). Under high-glucose conditions (diabetes), which is a risk factor for pancreatic cancer, curcumin suppressed EGF levels and activation of EGFR and ERK, resulting in reduced invasive ability and inhibition of metastatic-related factors (Li W. et al., 2019). Expression of COX-2, EGFR and *p*-ERK1/2 was also suppressed by curcumin in pancreatic adenocarcinoma cells similar to what was observed in lung adenocarcinoma cells (Lev-Ari et al., 2006). Furthermore, curcumin treatment for 24 h was found to decrease the expression of VEGFR1 and VEGFR2 in HUVECs (Fu et al., 2015). Human umbilical vein endothelial cells (HUVECs) are commonly used to understand tumour angiogenesis due to their major role in vascular homeostasis. Phosphorylation of ERK was also reduced reflecting the ability of curcumin to inhibit growth and migration of endothelial cells via blocking VEGFRs and downstream MAPK signaling pathway. Curcumin also reduced COX-2 expression in VEGF-activated human intestinal microvascular endothelial cells (HIMECs) via inhibition of phosphorylation of MAPK pathway components like p44/42 MAPK, p38 MAPK, and JNK (Binion et al., 2008). In oral cancer, curcumin upregulates the expression of insulin-like growth factor binding protein-5 (IGFBP-5) by increasing the nuclear expression of CCAAT/enhancer-binding protein α (C/EBPα), which is a transcriptional regulator of IGFBP-5 (Chang et al., 2010). This upregulation of IGFBP-5 mediated by activation of p38 by curcumin allowed it to bind to IGF, limiting the activation of IGF-1R and suppressing oral carcinogenesis. Meanwhile, a combination of curcumin and cetuximab decreased levels of phosphorylated EGFR, ERK, JNK, and surprisingly p38 in cisplatin-resistant oral cancer cells which contrasts the curcumin-mediated p38 activation observed by Chang et al. (2010). Accordingly, it has been found that curcumin differentially activates/inhibits p38 in different cancers (Watson et al., 2010; Wang et al., 2013; Tung et al., 2016); however, further research is required to elucidate how RTKs fit in this process. Curcumin treatment also abrogated HGF-induced epithelial-mesenchymal transition (EMT) in oral squamous cell carcinoma and prostate cancer cells by reducing levels of phosphorylated c-Met (HGFR) and inhibiting ERK activation (Hu et al., 2016; Ohnishi et al., 2020). Furthermore, levels of *p*-ERK, VEGF, and HIF-α were reduced by curcumin in liver cancer cells the same way they were reduced in IGF-1R-knockout liver cancer cells, suggesting that curcumin suppresses tumour progression in an IGF-1R-dependent manner involving the MAPK pathway (Chen et al., 2018). Curcumin also stimulated the expression of PPARγ by interrupting EGF and PDGF signaling in rat hepatic stellate cells (Zhou et al., 2007). This interruption involves repressing phosphorylation of PDGFR-β and EGFR and also reducing *p*-ERK and *p*-JNK. This is the second study observing the effects of curcumin on RTKs through PPARγ activation mediated by MAPK-inhibition.

#### 3.1.2 Phosphoinositide 3-Kinase/Akt/Mechanistic Target of Rapamycin

The phosphoinositide 3-kinase (PI3K)–Akt pathway is an ubiquitous signaling network that regulates growth, metabolism, biosynthesis of macromolecules and cellular homeostasis. It is mainly activated by growth factors, insulin, and cytokines. There are three classes of PI3K enzymes, however only class I PI3Ks are involved in cancer (Zhao and Vogt, 2008). Class I PI3Ks have four different isoforms (p110α, β, γ, and δ) which are encoded by *PIK3CA, PIK3CB, PIK3CG,* and *PIK3CD* (Fruman et al., 2017). Under normal physiological conditions, PI3K activation at the plasma membrane is followed by phosphorylation of phosphatidylinositol 4, 5-bisphosphate (PtdIns(4,5)P2) $\left(PIP_{2}\right)$ to produce phosphatidylinositol 3,4,5-trisphosphate (PtdIns(3,4,5)P3) $\left(PIP_{3}\right)$ which acts as a second messenger (Hoxhaj and Manning, 2020). $PIP_{3}$ then acts as a docking site and recruits proteins processing the pleckstrin homology (PH) domain such as the serine-threonine kinase, Akt. There are three isoforms of Akt (Akt1, Akt2, and Akt3) and once bound to $PIP_{3}$ , it is phosphorylated by phosphoinositide-dependent protein kinase 1 (PDPK1/PDK1) and mechanistic target of rapamycin (mTOR) complex 2 (mTORC2), increasing its activity (Fruman et al., 2017). Activated Akt phosphorylates many downstream substrates namely three critical proteins which are tuberous sclerosis complex 2 (TSC2), glycogen synthase kinase 3 (GSK3) and the forkhead box O (FOXO) transcription factors (TFs). Phosphorylation of TSC2 leads to activation of mTORC1 while phosphorylation of GSK3 leads to proteasomal degradation of several TFs like MYC, SREBP, nuclear factor erythroid 2-related factor 2 (NRF2), and HIF1α (Hoxhaj and Manning, 2020).

In cancer, there are four common genetic events that drive cancer progression which include 1) *PIK3CA* activating mutations, 2) PTEN loss-of-function mutations and deletions, 3) gain-of-function mutations in Akt-encoding genes, and lastly 4) amplification of RTKs that activate PI3K signaling (Hoxhaj and Manning, 2020). The PI3K pathway also plays a role in the control of glucose metabolism whereby constitutive activation of Akt promotes aerobic glycolysis and increased glucose uptake through GLUTs in cancer cells (Elstrom et al., 2004; Wieman et al., 2007). It also drives anabolic metabolism in excessively proliferating cells via promoting *de novo* lipid, nucleotide, and protein synthesis (Faridi et al., 2003; Porstmann et al., 2005; Saha et al., 2014). Moreover, the PI3K-Akt pathway has been found to trigger ROS-producing processes as well however more research in needed to elucidate the exact downstream pathways involved in ROS production in cancer cells (Chen et al., 2003; Hoxhaj and Manning, 2020). Activation of RTKs and downstream PI3K signaling have been implicated in acceleration of tumour growth, malignant transformation, and resistance in different cancers (Zhang et al., 2007; Jung et al., 2015; Starska et al., 2018).

In lung cancer, curcumin has been shown to downregulate EGFR expression by inducing expression of an E1-like ubiquitin-activating enzyme, UBE1L, which represses EGFR protein expression and promotes EGFR internalization (Jiang et al., 2014). Curcumin also reduced Akt phosphorylation and repressed the EGFR/Akt pathway through UBE1L induction. Levels of PI3Kwere also reduced in lung cancer cells following curcumin treatment, which is likely one of the pathways that resulted in the reduced VEGF expression that was also observed (S.-S. Lin et al., 2009). Furthermore, curcumin pre-treatment of lung cancer cells decreased the HGF-induced phosphorylation of c-Met and downstream PI3K signaling components like Akt, mTOR, and S6, leading to EMT inhibition (Jiao et al., 2016).

In colon cancer, curcumin combined with 5-fluorouracil (5-FU) and oxaliplatin (FOLFOX) were found to induce higher levels of apoptosis by reducing both the expression and activation of EGFR, IGF-1R, HER-2, and HER-3 by a greater magnitude then either agent alone (Patel et al., 2008). An analysis of downstream signaling components also found downregulation of expression and activation of Akt and COX-2 after curcumin and FOLFOX combination treatment. COX-2 activation and expression has been linked to Akt phosphorylation in several cancers, hence curcumin may mediate COX-2 activation by targeting RTKs and the downstream PI3K/Akt pathway (St-Germain et al., 2004; Glynn et al., 2010). Similar results were also achieved when curcumin was used to treat FOLFOX-surviving colon cancer cells, highlighting the importance of EGFR/IGF-1R/Akt signaling inhibition by curcumin in chemo-resistant cells (Patel et al., 2010). A combination of curcumin and dasatinib also resulted in the downregulation of the EGFR/IGF-1R/Akt axis, further providing evidence that this may be one of the primary mechanisms of inhibition by curcumin either alone or in synergistic combination with other anti-cancer agents (Nautiyal et al., 2011a). Gene expression analyses carried out on colon cancer cells lines found upregulation of many genes including VEGF, FGFR1, and Akt after exposure to high concentration of curcumin which contrasts the usual downregulation, however it could be toxic-related effects of the fairly high concentration of curcumin used as stated before (Van Erk et al., 2004).

In breast cancer, it was found that curcumin combined with herceptin (trastuzumab) was effective against herceptin-resistant breast cancer cells, likely mediated by the decreased levels of HER-2 oncoprotein and phosphorylated Akt (Lai et al., 2012). In another study, curcumin also inhibited the basal phosphorylation of Akt/PKB in breast cancer cells but not directly, suggesting that it could be due to the decrease in EGF-induced EGFR activation that was observed (Squires et al., 2003). The study by Borah at et al. (2020) aimed to inhibit the Hh/Gli-EGFR signaling pathway in breast cancer by co-delivering curcumin and a Hh/Gli small molecule antagonist GANT61 via polymeric nanoparticles. Based on immunofluorescence studies, they found that the GANT61-curcumin PLGA NPs managed to decrease EGFR protein expression and also PI3K expression, which may contribute to the inhibitory migration potential of breast adenocarcinoma cells.

In liver cancer, curcumin decreased VEGF, PI3K, and Akt expression, with one study suggesting that this effect is mediated via curcumin-inhibition of IGF-1R to suppress angiogenesis (Chen et al., 2018; Pan et al., 2018). Curcumin also reduced the phosphorylated PI3K/Akt levels by inhibiting tyrosine phosphorylation of PDGFRβ and EGFR, which led to activation of PPARγ and subsequent induction of apoptosis (Zhou et al., 2007). It was found that a combination of curcumin and metformin significantly reduced PI3K, *p*-Akt, and *p*-mTOR while also significantly increasing expression of PTEN, which is a negative regulator of the PI3K pathway (Zhang et al., 2018). Combination treatments for curcumin and β-phenylethyl isothiocyanate (PEITC) as well as curcumin and docetaxel were found to decrease EGFR expression and activation, PI3K expression, and *p*-Akt which led to enhanced apoptosis and reduced cell proliferation in prostate cancer cells (Kim et al., 2005; Banerjee et al., 2017). Furthermore, in hyperglycemic-pancreatic and oral cancer cells, curcumin was found to reduce cell proliferation by inhibiting the EGF/EGFR/Akt pathway (Zhen et al., 2014; Li W. et al., 2019). Besides, curcumin also decreased gene expression of EGFR and expression of PI3K (p110α), Akt, and mTOR in tongue and hypopharynx squamous cell carcinoma (SCC), highlighting the therapeutic potential of curcumin-mediated RTK inhibition in preventing head and neck cancer progression (Borges et al., 2020). Meanwhile, another study chemically induced skin carcinogenesis in transgenic mice overexpressing IGF-1 and found that a curcumin diet significantly reduced tumour multiplicity, tumour size, and cell proliferation (Kim et al., 2014). The underlying mechanism leading to these effects was curcumin-mediated inhibition of IGF-1R, insulin receptor substrate-1 (IRS-1), Akt, S6K, and eukaryotic translation initiation factor 4E-binding protein 1 (4EBP1) phosphorylation in a dose-dependent manner (Kim et al., 2014). Lastly, curcumin downregulated phosphorylation of PI3K-p85, Akt, mTOR, and further downstream effectors 4EBP1 and S6K in bladder cancer. Interestingly, IGF-1 knockdown did not alter the inhibitory effects of curcumin, suggesting that curcumin mainly acts through the IGF-2/IGF-1R pathway and downstream PI3K signaling in bladder cancer (Tian et al., 2017).

#### 3.1.3 Janus Kinase/Signal Transducers and Activators of Transcription

The Janus kinase (JAK)–signal transducer of activators of transcription (STAT) pathway is one of the pathways involved in RTK signal transduction and it can be activated by diverse cytokines, interferons and other related components. It allows direct communication from membrane-to-nucleus through the interaction between four Janus kinases (JAKs)—JAK1, JAK2, JAK3, and TYK2, and seven signal transducers and activators of transcription (STATs)—STAT1, STAT2, STAT3, STAT4, STAT5a, STAT5b, and STAT6 (O'Shea et al., 2015). Once a ligand binds to the receptor, receptor-associated JAKs are activated and they proceed to cross-phosphorylate each other and also the intracellular tail of their receptors. This creates a docking sites for the recruitment of cytoplasmic STATs. STATs are then activated via JAK-phosphorylation and they translocate to the nucleus to regulate gene expression by binding DNA (O'Shea et al., 2015). Many RTKs engage with the JAK/STAT pathway to promote proliferation and differentiation (Boccaccio et al., 1998; Andl et al., 2004; Masamune et al., 2005).

In cancer, constitutive JAK/STAT activation normally occurs through increased expression of ligands and activating mutations of receptors, JAKs, or STAT themselves (O'Shea et al., 2015). JAK mutations have been found widely in leukemia and many solid tumours that contribute to cancer cell migration, proliferation, and invasion (Walters et al., 2006; Jeong et al., 2008; Stelloo et al., 2016; Xu et al., 2017). STAT3 and STAT5 are also commonly mutated a variety of cancers. Mechanisms leading to their constitutive activation include lack of negative regulation, somatic mutations causing hyperactivation, overstimulation, positive feedback loops and crosstalk with other signaling pathways leading to resistance, poor prognosis, tumour progression, and worse overall survival (Zhang and Lai, 2014; Halim et al., 2020). However, studies have shown that both STAT3 and STAT5 have tumour suppressor roles, reflecting their paradoxical nature (Igelmann et al., 2019). RTKs have also been shown to promote cancer progression and tumour immunosuppression via JAK/STAT pathways (Su et al., 2018; Li P. et al., 2019; Song et al., 2020).

Curcumin was found to inhibit expression of phosphorylated STAT3, JAK1 JAK2, and JAK3 in SCLC cells (Yang et al., 2012). Levels of VEGF were also downregulated after curcumin treatment however this study focused on IL-6-dependent STAT3 activation, hence it is not certain if curcumin mediated inhibition via the RTK signaling pathway. In laryngeal squamous cell carcinoma, curcumin inhibited the expression of JAK2 and phosphorylation of STAT3, which is JAK2-dependent (Hu et al., 2014). Curcumin also inhibited VEGF mRNA and protein expression via the downregulation of this JAK2/STAT3 pathway which likely reduced VEGF-induced activation of VEGFR. However, there is only a handful of studies linking the effects of curcumin to RTKs and the JAK/STAT pathway as most studies only examine how curcumin inhibits JAK/STAT directly or via other signaling pathways.

The available literature on curcumin and JAK/STAT in cancer mainly look at how curcumin inhibits phosphorylation of various JAKs and namely STAT3 and STAT5. In blood cancers, a number of studies found that curcumin downregulated phosphorylation of JAK2, JAK3, TYK2, STAT3, STAT5a, and STAT5b (Rajasingh et al., 2006; Park et al., 2008; Petiti et al., 2019). Meanwhile, it was found that curcumin did not affect the phosphorylation of STAT proteins in chronic leukemia cells but only decreased their nuclear expression (Blasius et al., 2006). Curcumin also reduces migration, proliferation, and invasion directly by modulating levels of phosphorylated JAKs and STATs or indirectly by regulating protein inhibitors of activated STAT-3 (PIAS-3), suppressors of cytokine signaling (SOCS3), and miRNAs involved in JAK/STAT activity in a range of cancers including eye, ovarian, and endometrial cancers (Saydmohammed et al., 2010; Li Y. et al., 2018). Inhibition of the JAK/STAT pathway by curcumin in esophageal cancer cells also increased cell adhesion which is normally reduced in cancer (Zheng et al., 2018). Combination treatment of curcumin and cisplatin managed to inhibit phosphorylation of JAK and STAT3 in ovarian and papillary thyroid cancer cells leading to enhanced proliferation and reduced stemness of potential cancer stem cells (Khan AQ. et al., 2020; Sandhiutami et al., 2021). In osteosarcoma cells, curcumin inhibited the *p*-JAK2/p-STAT3 pathway which was involved in lung metastasis whereas in lung cancer, curcumin suppressed activation of p-STAT3 both *in vitro* and *in vivo* (Alexandrow et al., 2012; Sun et al., 2019). Several studies also examined the potency of curcumin analogues, FLLL31 and FLLL32, which were designed to specifically bind to JAK2 and STAT3 SH2 domains (Lin et al., 2010b). Both analogues were found to effectively suppress *p*-JAK2 and p-STAT3 and also key apoptotic proteins (Lin et al., 2010a; Lin et al., 2010b; Abuzeid et al., 2011). FLLL32 however showed very little inhibition of RTKs like EGFR, HER2 and Met (Lin et al., 2010b). Another curcumin analogue, L48H37, also decreased the phosphorylation of JAK1, JAK2, JAK3, and STAT3 in osteosarcoma cells (Lu et al., 2020). Gene expression profiling and recent RNA sequencing technology also identified both upregulation and downregulation of JAK/STAT signaling pathway components, with some of these studies also documenting changes in RTK gene expression (Van Erk et al., 2004; Teiten et al., 2009; Zhao W. et al., 2015). Further studies are needed to clarify the link between curcumin, RTKs and the JAK/STAT pathway.

#### 3.1.4 Nuclear Factor Kappa B

The nuclear factor kappa B (NF-κB) consists of a group of transcription factors (TFs) that are responsible for many biological processes like inflammation, cell proliferation, immunity and apoptosis (Zinatizadeh et al., 2021). This family of TFs include five proteins which are RelA, RelB, c-Rel, p100, and p150. These proteins possess the rel homology domain (RHD) that assists in dimerization, binding to DNA and interaction with specific inhibitors (Zinatizadeh et al., 2021). Inhibitors of NF4EBP-κB are from the IκB inhibitor family comprising of IκBα, IκBβ, and IκBε. The association between NF-κB and IκBs form dimers that are retained in the cytoplasm in an inactive state (Dolcet et al., 2005). The phosphorylation of IκBs by IκB-kinases (IKKs) leads to IκB degradation and NF-κB liberation. NF-κB then enters the nucleus and regulates the transcription of a wide array of genes that code for growth factors, cytokines, cell adhesion molecules, pro- and anti-apoptotic proteins (Luo et al., 2005). NF-κB activation can be caused by a variety of signaling pathways including activation of Ras/MAPK, PI3K/Akt, and JAK/STAT which are commonly mediated by RTKs (Dolcet et al., 2005; Zhang et al., 2021).

In cancer, NF-κB activation can lead to apoptosis resistance through the expression of inhibitors of apoptosis (IAPs), members of anti-apoptotic Bcl-2 family and also proteins that disrupt the death receptor apoptotic pathway (Wang et al., 1998; Catz and Johnson, 2001; Kreuz et al., 2001). NF-κB activity also enhances cell cycle progression by inducing expression of key cell cycle proteins like cyclin D1 and invasion-related proteins like matrix metalloproteinases (MMPs) as well as VEGF and COX-2 that are important in tumour growth (Dolcet et al., 2005; Li et al., 2011; Li et al., 2016). Constitutive NF-κB activation has been observed in 66% of colorectal cancer cell lines whereas activating NF-κB mutations commonly occur in hematopoietic tumours (Hassanzadeh, 2011; Xia et al., 2014). Generally, mutations in upstream signaling molecules like MAPK proteins or RTKs themselves lead to constitutive activation of NF-κB in solid tumours (Tilborghs et al., 2017). Similar to many other proteins, NF-κB can act as a tumour promoter or tumour suppressor under different circumstances. As a tumour growth promoter, NF-κB has been found to induce expression of oncogenic microRNAs, promote expression of immune checkpoint proteins like PD-L1 and also act in sync with STAT3 and AP-1 to induce tumour-associated inflammation (Galardi et al., 2011; Asgarova et al., 2018; Ji et al., 2019). Meanwhile, loss or inhibition of NF-κB has been found to increase immortalization of cells and invasion, reflecting its tumour suppressive functions (Vandermark et al., 2012; O’Reilly et al., 2018). Deregulation of RTK/NF-κB signaling has been observed in various cancers (Matušan-Ilijaš et al., 2013; Spirina et al., 2017; Lai et al., 2018).

Curcumin in combination with herceptin decreased levels of NF-κB in a dose-dependent manner in HER-2-overexpressed breast cancer cells, overcoming herceptin resistance (Lai et al., 2012). Curcumin also suppressed osteopontin (OPN)-induced VEGF expression (Chakraborty et al., 2008). OPN is one of the main markers of breast cancer progression. Further analysis found that curcumin inhibited NF-κB activation which led to suppression of OPN-induced VEGF (Chakraborty et al., 2008). This suggests that curcumin may inhibit the VEGF/VEGFR signaling via NF-κB inhibition. In lung cancer, *in vivo* mice studies also showed that curcumin regulated tumour angiogenesis by decreasing VEGF expression through NF-κB inhibition (Li X. et al., 2018). Two separate studies examined combinations of curcumin with dasatinib and EGF-Receptor Related Protein (ERRP) in colon cancer. Both studies found that curcumin inhibited EGFR, IGF-1R, and NF-κB activity and this effect was more pronounced with combination treatments (Reddy et al., 2006; Nautiyal et al., 2011a). Moreover, curcumin analogues, EF31, and UBS109, were found to induce downregulation of VEGF, HIF-α, and COX-2 as well as inhibit IKKs, NF-κB translocation and NF-κB DNA binding based on *in vitro* and *in vivo* cancer studies (Olivera et al., 2012; Nagaraju et al., 2015; Rajitha et al., 2017). However, there is still a lack of studies looking into how curcumin modulates NF-κB via RTK signaling pathways or vice versa.

Similar to the JAK/STAT pathway, a range of studies have examined the direct effect of curcumin on NF-κB signaling. Curcumin was found to inhibit both NF-κB and Wnt signaling in cervical cancer while it also inhibited AP-1, NF-κB, and HPV E6 proteins in HPV-positive oral carcinoma, abolishing HPV transcription (Mishra et al., 2015; Ghasemi et al., 2019). Meanwhile, a phase I/II study on patients with multiple myeloma found that orally administered curcumin had no serious adverse effects and also reduced constitutive NF-κB activation (Vadhan-Raj et al., 2007). Furthermore, curcumin and its analogues have also been combined with cytotoxic drugs like cisplatin and doxorubicin and they were found to downregulate the drug-induced increase of NF-κB in liver and breast cancer (Notarbartolo et al., 2005; Meiyanto et al., 2014). Other curcumin combinations involving tolfenamic acid and Chinese goldthread also inhibited cell proliferation via disruption of NF-κB translocation into the nucleus and NF-κB transcriptional activity respectively (Zhao et al., 2014; Basha et al., 2016).

## 4 Curcumin-Receptor Tyrosine Kinase Inhibitor Combination

Tyrosine kinase inhibitors (TKIs) are a form of targeted therapy that interfere with the activity of oncogenic tyrosine kinases (TKs). Some of their inhibitory mechanism include competing with ATP for binding sites on the catalytic domain of TKs and decreasing phosphorylation of TKs which lead to inhibition of tumour cell repair, induction of apoptosis, and blockage of G1 phase cell division (Jiao et al., 2018). These small molecule inhibitors are orally active, safe, and effective in tumour inhibition (Arora and Scholar, 2005). As of 2019, the Food and Drug Administration (FDA) has approved 48 protein kinase inhibitors of which 25 target receptor tyrosine kinases (Roskoski, 2019). RTKIs can either be single-targeted or multi-targeted. Single-targeted RTKIs include common ones like gefitinib, erlotinib, and lapatinib that inhibit EGFR and axitinib and lenvatinib that target VEGFR while multi-tyrosine kinase inhibitors include imatinib, sorafenib, sunitinib, pazopanib, and regorafenib which target a mix of RTKs and non-RTKs (Jiao et al., 2018). Certain aspects need to be taken into account when deciding whether to use multiple single kinase inhibitors or a single multi-kinase inhibitor and these include aspects involving efficacy, pharmacokinetics, tumour microenvironment, and resistance (Broekman et al., 2011).

As with the use of most drugs, RTKI use is often followed by the rise of resistance. Mechanisms of resistance against RTKIs include mutations, gene amplification, and RTK overexpression, overexpression of downstream kinases, increased expression of drug efflux pumps, and gene fusion, most of which were mentioned in the section regarding oncogenic RTKs (Broekman et al., 2011; Jiao et al., 2018). RTKI resistance can be either primary (intrinsic) or secondary (acquired) whereby primary resistance is when there is a lack of tumour response to treatment while secondary resistance involves exposure to the RTKI and subsequent selection of resistant tumour cells (Pottier et al., 2020). As a result, combination treatments are becoming the preferred regimen to treat cancers. Many studies are examining the combination RTKIs with chemotherapy drugs, immunotherapy, and radiotherapy (Goldberg et al., 2013; Liang et al., 2018; Khan M. et al., 2020). This in turn presents a new challenge of finding a positive balance between the toxicities caused by increased drug administration and survival benefits. Curcumin is being explored as viable solution to overcome this challenge and can possibly serve as a substitute for certain drugs in combination treatments. A compelling reason for the use of curcumin in drug combinations is that its low toxicity allows doses of up to 12 000 mg a day which are well tolerated in humans (Basnet and Skalko-Basnet, 2011). The combination of curcumin and anti-cancer drugs like RTKIs, can remove a large portion of toxicity induced when two conventional drugs are combined, and indeed studies have found curcumin to reduce chemotherapy- and radiotherapy-induced side effects (Mansouri et al., 2020). Findings from a range of curcumin-RTKI combination studies are summarized in Table 2 and will be further reviewed in the following sections.

**TABLE 2:** Summary of curcumin-RTKI combination studies.

Treatment	Cancer	Molecular targets/Pathways		Ref
		*In vitro*	*In vivo*	
Curcumin + gefitinib	Lung	↓ EGFR	↓ EGFR Akt, c-MET, cyclin D1 and PCNA, ↑ caspase-8, -9, PARP, p38 activation	Lee et al. (2011)
		↓ EGFR/p-EGFR, Akt/p-Akt protein, ↓ mRNA and protein levels of AXL, HLJ1 and MMP, ↑ G-actin/F-actin ratio	—	Lee et al. (2007)
		↓ p38, ERK1/2 and Akt phosphorylation	—	Xin et al. (2017)
		↓ EGFR activity via inhibiting binding of HDAC1 to Sp1, ↓ EGFR, c-MET, Her-2, AXL and IGF-1R	↓ Sp1, HDAC1, EGFR, survivin and ↑ LC3, Beclin 1 and cleaved caspase-3	Chen et al. (2019)
	Oral	↓ MMP, ↑ caspase-3 and -7, AIF	↑ caspase-6, -7, Beclin 1, Bcl-2 and *p*-EGFR	Hsiao et al. (2018)
		Beclin 1, ATG5, LC3, p62/SQSTM, ULK1, VPS34		
		↑ PARP, cytochrome C, p53, caspase-9 and -3, ↓ XIAP	—	Lai et al. (2019)
Curcumin + erlotinib	Lung	↓ EGFR, *p*-EGFR, survivin, p-p65 (NF-κB), ↑ cleavage of caspase-3, -9 and cytochrome c release	—	Li et al. (2013)
		↑ ikappaB	↑ ikappaB, ↑ NF-κB	Yamauchi et al. (2014)
	Pancreatic	↑ PDK4, ↓ α_V_β_3_ integrin	—	Javadi et al. (2018)
Curcumin + lapatinib	Breast	↑ E-cadherin, ↓ Snail, vimentin, N-cadherin, CD44, ALDH1, ABCG2, SOX2	—	Liu et al. (2015)
		↓ p-Her2, *p*-Akt, total Her2	—	Saxena et al. (2020)
Curcumin + sorafenib	Liver	↓ MMP	—	Cao et al. (2015)
		↓ cyclin D1	—	Hosseini et al. (2019)
		↑ TIMP-1, ↓ MMP-9, p65, *p*-ERK1/2, CD133	↑ TIMP-1, ↓ MMP-9, p65, *p*-ERK1/2, CD133	Hu et al. (2015)
		↓ MMP, p27, cyclin A2, cyclin B, cyclin D1, p-Rb, Bcl-xL, ↑ Bax, cleaved caspase-3 and -9	—	Bahman et al. (2018)
		—	↓ ALT, MDA, vimentin, IL-1β, NF-κB, *p*-JAK1/2, p-STAT3, HIF-α, LDH, TG, FASN, lactate, D-fructose, D-glucose, hexadecanoic acid,CPT1A, *p*-Akt, ↑ CD4^+^ T cells, NK cells, E-cadherin, IL-4, HDL-C, apoA1, p53	Man et al. (2020)
	Thyroid	↓ *p*-ERK, *p*-Akt	—	Zhang et al. (2016)
	Renal	↓ Rb	—	Debata et al. (2013)
Curcumin + sunitinib	Renal	↓ p-Rb, cyclin D1	—	Debata et al. (2013)
Curcumin + regorafenib	Colorectal	↑ cleaved caspase-3 and LC3-II	—	Su and Wu, (2017)
		↑ cleaved PARP, ↓ *p*-MEK, *p*-ERK	—	Wu et al. (2019a)

Abbreviations: PCNA: Proliferating cell nuclear antigen; PARP: Poly (ADP-1144 ribose) polymerase; MMP: Matrix metalloproteinase; HDAC1: Histone deacetylase 1; LC3: Microtubule-associated protein 1A/1B-light chain 3; AIF: Apoptosis inducing factor; ATG5: Autophagy related 5; SQSTM: Sequestosome; ULK1: Unc-51 like autophagy activating kinase; VPS34: Vacuolar protein sorting 34; Bcl-2: B-cell lymphoma 2; XIAP: X-linked inhibitor of apoptosis protein; PDK4: Pyruvate dehydrogenase (acetyl-transferring) kinase isozyme 4; ALDH1: Aldehyde dehydrogenase 1; ABCG2: ATP-binding cassette super-family G member 2; SOX2: SRY (sex determining region Y)-box 2; TIMP1: Tissue inhibitor of metalloproteinase 1; Rb: Retinoblastoma protein; Bcl-xL: B-cell lymphoma extra large; Bax: Bcl-2-associated X protein; ALT: Alanine aminotransferase; MDA: Malondialdehyde; IL-1β: Interleukin 1 beta; HIF-α: Hypoxia-inducible factor 1-alpha; LDH: Lactate dehydrogenase; TG: Triglyceride; FASN: Fatty acid synthase; CPT1A: Carnitine palmitoyltransferase 1A; NK: Natural killer cells; IL-4: Interleukin 4; HDL-C: High density lipoprotein cholesterol; apoAI: Apolipoprotein A Ⅰ.

### 4.1 Curcumin and Single-Targeted RTKIs

Curcumin has been studied in combination with a few single-targeted RTKIs mainly EGFR TKIs like gefitinib, erlotinib and lapatinib. In a gefitinib-resistant lung cancer cell line (H1975), a combination of 15 µM of curcumin and 1 µM of gefitinib was found to have the same antiproliferative effect as 20 µM of gefitinib (Lee et al., 2011). In addition to EGFR, c-Met and Akt reduction, this study also found that combination treatment significantly lowered tumour growth on xenograft mice models and more importantly, 60 mg/kg of gefitinib combined with 1 g/kg of curcumin showed comparable results to 120 mg/kg of gefitinib. Side effects of gefitinib like villi damage and gastrointestinal effects were also attenuated by curcumin (Lee et al., 2011). Several other curcumin and gefitinib combination studies also found that curcumin promotes the inhibitory activity of gefitinib through downregulation of EGFR, MAPK, and PI3K signaling pathways (Lee et al., 2007; Xin et al., 2017; Chen et al., 2019). It was further found that curcumin and gefitinib also suppressed Sp1-and HDAC-induced EGFR transcription which led to induction of autophagy (Chen et al., 2019). In human oral cancer SAS cells, curcuminoids (curcumin, demethoxycurcumin or bisdemethoxycurcumin) combined with gefitinib induced certain apoptotic and autophagic proteins and overall led to higher levels of cell death compared to each agent alone (Hsiao et al., 2018). Meanwhile, further *in vivo* analysis showed that gefitinib combined with curcumin and demethoxycurcumin greatly decreased tumour volume in mice. Curcumin and gefitinib-loaded nanoparticles (NPs) have also been tested in oral cancer SAS cells (Lai et al., 2019). These γ-PGA-Gef/Cur NPs induced cell death through caspase and mitochondria-dependent pathways and low doses of Gef/Cur loaded NPs significantly decreased tumour weight as well. Meanwhile, several curcumin, and erlotinib combination studies were also found to strongly inhibit tumour growth and decrease tumour weight in xenograft mice models (Li et al., 2013; Yamauchi et al., 2014). Co-administration of curcumin and erlotinib was found to reduce cell viability of lung cancer cells via ikappaB elevation (Yamauchi et al., 2014). Additionally, it was also found that a relatively lower dose of curcumin also sensitized erlotinib-resistant NSCLC cells to erlotinib’s cytotoxic effects, reduced expressions of EGFR and also inhibited NF-κB activation (Li et al., 2013). A few studies also employed the use of nano-based delivery systems to combine curcumin and erlotinib. A combination of curcumin and erlotinib-loaded Methoxypoly (ethylene glycol) Poly (caprolactone) (Mpeg-pcl) was found to increase PDK4 gene and decrease αvβ3 integrin expression in colorectal cancer cells (Javadi et al., 2018). These components are involved in erlotinib-resistance, and the addition of curcumin to erlotinib treatment seems to influence associated drug resistance signaling pathways. An erlotinib and curcumin conjugated carrier-free nanoassembly (EPC) was also developed and found to have better tumour-penetrating and anti-migratory properties in addition to the absence of systemic toxicity (Cheng et al., 2020). Lastly, combinations of curcumin and lapatinib were also found to increase lapatinib-induced inhibition of the Her2-Akt pathway, reverse lapatinib resistance and decrease metastatic potential in breast cancer cells (Liu et al., 2015; Saxena et al., 2020). Currently, only one phase I clinical trial has been conducted investigating the combination treatment of curcumin and EGFR-TKIs (gefitinib and erlotinib) (Esfahani et al., 2019). An enhanced bioavailable curcumin formulation was administered together with gefitinib or erlotinib. Overall, no evidence of toxicity was observed and adverse effects, if any, were pre-existing due to TKI therapy. Curcumin was found to improve the quality of life and appeared to be a safe adjuvant to TKI therapy.

### 4.2 Curcumin and Multi-Targeted RTKIs

Recently, there has been an increase in the number of studies investigating the *in vitro* combinatorial effects of curcumin and multi-kinase inhibitors, namely sorafenib, sunitinib, and regorafenib. Sorafenib is an orally administered pyridine multi-kinase inhibitor (MKI) that inhibits RTKs like VEGFR, PDGFR, RET, and MAPK signaling components like Raf-1, Braf, Braf mutants, and c-Kit (Wilhelm et al., 2004; Di Gion et al., 2011). As of now, combination treatment of sorafenib and curcumin has mostly been studied in hepatocellular carcinoma (HCC), with most of them involving nanoparticle (NP)-based delivery. These delivery systems include directed self-assembly NPs, pH-sensitive lactosylated NPs, polymeric nanoparticle formulations of curcumin and nanomicelles (Cao et al., 2015; Hu et al., 2015; Hosseini et al., 2019; Bian and Guo, 2020). All these studies found that an NP-based combination of curcumin and sorafenib showed higher cytotoxicity and induced higher apoptosis in HCC than either one alone. Some of them also demonstrated enhanced anti-angiogenic effects (Cao et al., 2015), improved *in vivo* tissue distribution (Cao et al., 2015; Bian and Guo, 2020), good tolerance (Bian and Guo, 2020), and downregulation of biomarkers/genes involved in cancer progression (Hu et al., 2015). Free drug combination treatments of curcumin and sorafenib also showed promising results like increased apoptosis, disruption of cell cycle progression, and protection of liver function from sorafenib-induced effects (Bahman et al., 2018; Man et al., 2020). This combination also remarkably increased the proportion of CD4^+^ T cells and natural killer cells and inhibited sorafenib-induced EMT via downregulation of JAK/STAT and NF-κB pathway proteins (Man et al., 2020). MAPK and PI3K pathway components were also reduced by curcumin and sorafenib in thyroid cancer cells, decreasing migration and invasion (Zhang et al., 2016).

Sunitinib is a pyrole multi-kinase inhibitor that mainly inhibits VEGFR and PDGFR, and it is also a first-generation MKI like sorafenib (Mendel et al., 2003; Di Gion et al., 2011). There are only a few studies that have examined curcumin-sunitinib combinations. Combinations of curcumin with erlotinib, sorafenib, and sunitinib were studied in breast cancer cells and it was found that curcumin combined with sunitinib exhibited the highest reduction of cell viability (Chen et al., 2016). This combination was brought forward into *in vivo* analysis, and the efficacy of combination treatment was higher than mono-therapy; however, no statistical significance was achieved. In addition, bovine serum albumin (BSA)-encapsulated curcumin and sunitinib was found to be more effective than this free drug combination (Chen et al., 2016). These findings inspired an additional study whereby curcumin and sunitinib were co-loaded into BSA-supermagnetic iron oxide nanoparticles (SPIOs) (Chen et al., 2017). This formulation showed the highest amount of tumour inhibition and simultaneously the least amount of toxicity while also efficiently delivering the drugs to the tumour site based on *in vitro* and *in vivo* breast cancer models. In renal cancer cells, curcumin combined with sunitinib decreased the IC50 of sunitinib by four-fold; however, this effect was not observed with sorafenib (Debata et al., 2013). This suggests that the therapeutic dose of sunitinib can be reduced when combined with suitable concentrations of curcumin.

Regorafenib is one of the newer orally active MKIs mainly targeting VEGFR and PDGFR (Strumberg and Schultheis, 2012). As such, only two studies have investigated the combination treatment of curcumin and regorafenib in colorectal cancer cells (Su and Wu, 2017; Wu CS. et al., 2019). Curcumin appeared to act like a MEK inhibitor and most likely targets other genes as well, producing a synthetic lethal effect in KRAS-mutant colorectal cancer cells (Wu CS. et al., 2019). The combination of curcumin and regorafenib only showed additive/synergistic effects in KRAS-mutant and not KRAS-wildtype cells, suggesting their possible use in the treatment KRAS-mutant colorectal cancer. Imatinib and dasatinib are first- and second-generation pyrimidine TKIs respectively, and are mainly known to be non-RTKIs,; however, both also target PDGFR (Natoli et al., 2010). Several studies have combined curcumin with both imatinib and dasatinib. Most of the studies found that curcumin enhanced the anti-leukemia effects of imatinib by downregulation of the Bcr/Abl gene (Bae et al., 2005; Gong et al., 2012; Guo et al., 2015). Nanostructured lipid carriers of curcumin and imatinib were also found to have superior effects than imatinib alone (Setareh and Jaleh, 2018; Varshosaz et al., 2021).

Furthermore, a case report stated that curcumin and imatinib successfully treated a patient having c-KIT-positive adenoid cystic carcinoma for the first time, whereby complete anatomic and metabolic response was observed after 24 months (Demiray et al., 2016). On the other hand, curcumin and dasatinib combination treatments have also shown reduced metastatic potential, regression of mice intestinal adenomas and decreased cancer stem cell populations in colon cancer cells (Nautiyal et al., 2011a; Nautiyal et al., 2011b). However, none of the studies combining curcumin and imatinib/dasatinib recorded modulations of PDGFR despite it being a known target of these two drugs. Most of these studies reported changes in downstream signaling pathway components which could possibly be due to upstream regulation of its known target, PDGFR.

## 5 Conclusion

Curcumin possesses many of the features required to be an ideal anti-cancer therapeutic agent, especially with its enigmatic ability to singularly target a legion of signaling molecules. Further studies revealed that curcumin targets RTKs and their downstream signaling pathways such as MAPK, PI3K/Akt, JAK/STAT, and NF-κB pathways which are involved in essential cellular processes like proliferation, apoptosis, cell cycle progression, and migration. Curcumin-mediated modulation of RTK expression or activation leads to positive outcomes like reduced proliferation, increased apoptosis, and decreased migration. .In many cases, the specific mechanism of action depends on the cellular environment and type of cancer.

Multiple studies have shown that curcumin can overcome resistance and enhance the apoptotic effects of existing TKI drugs. There are still many unanswered questions regarding how curcumin targets RTKs, especially whether or not direct binding occurs. Additional studies are also required to elucidate the effects of curcumin on RTKs along with changes in the JAK/STAT and NF-κB pathways. Many existing studies examine how curcumin targets RTKs or how curcumin targets specific pathways, however, an extensive analysis would require investigating all three components simultaneously (curcumin, RTKs, and signaling pathways) to obtainclearer understanding. In addition, it would be interesting to see how non-RTKs fit into this whole process since they make up many of the essential intracellular components. One of the main limitations of curcumin is its poor bioavailability in cellular environments. Various analogues of curcumin are being developed with superior bioavailability and improved anti-cancer properties. The use of nano-delivery systems is also gaining attention, especially in the delivery of curcumin and chemotherapy drugs. .There is a need for more *in vivo* and overall toxicity studies involving curcumin and its analogues. Combination treatments of curcumin and TKIs also need to be further studied to build a more substantial basis of evidence to ease curcumin progression into clinical trials. In conclusion, among the many mechanisms employed by curcumin, inhibition of receptor tyrosine kinases appears to be a significant element. It would be crucial to explore the implications for TKI therapy and whether the integration of curcumin and TKIs can improve treatment efficacy.
